# Reversed Doppler effect based on hybridized acoustic Mie resonances

**DOI:** 10.1038/s41598-020-58370-3

**Published:** 2020-01-30

**Authors:** Chen Liu, Houyou Long, Chen Zhou, Ying Cheng, Xiaojun Liu

**Affiliations:** 1 Key Laboratory of Modern Acoustics, Department of Physics and Collaborative Innovation Center of Advanced Microstructures, Nanjing University, Nanjing, 210093 China; 2 State Key Laboratory of Acoustics, Institute of Acoustics, Chinese Academy of Sciences, Beijing, 100190 China

**Keywords:** Metamaterials, Acoustics

## Abstract

The realization of reversed Doppler effects in double-negative acoustic metamaterials remains challenging. This paper demonstrates the reversed Doppler effect associated with sound wave propagation in negative group velocity in hybridized metamaterial (HM) system using a simple Mie-resonator configuration. Double-negative acoustic parameters act simultaneously on the effective dynamic bulk modulus and mass density within overlapped frequency region of multiple Mie resonances. Notably, while ordinary media exhibits higher received frequency during the approach and lower during the recession, we observe that in HM the detected signals show redshift compared to the emitted frequency when approaching to the source while depict blue shift when receding from the source. On this basis, the HM exhibits negative phase velocity with reversed wavefronts and negative refraction effect for certain frequency range. Focusing of sound waves emitted from a point source is further realized with a flat lens composed by such a HM slab.

## Introduction

The Doppler effect, referring to the change in detected frequency of waves for an observer moving relative to the source, has been widely used in various technologies including medical imaging, radar detection, astronomy and many more^1–3^. However, typical Doppler effect in ordinary medium exhibits higher received frequency (compared to the emitted frequency) during the approach and lower during the recession. The past decade has witnessed significant research efforts in exploring the artificial metamaterials enabling unusual wave properties and responses not found in nature, such as phase control^4–8^, wave-front modulation^9–11^ and topological acoustics^12,13^. As a consequence, interest in abnormal Doppler effect has reemerged. The reversed Doppler effect is theoretically proposed in an electromagnetic media with simultaneously negative permittivity and negative permeability^14,15^, since the group velocity and phase velocity are in the opposite directions. Thus, an approaching source decreases the frequency of received signal whereas a receding source increases it. Various demonstrations of the reversed Doppler effect have been observed in transmission line^16–19^ and photonic crystals^20,21^. For example, Ran *et al*. observed the reversed Doppler effect with an external radio-frequency source on an electronically reconfigurable transmission line^6^. Chen *et al*. reported the experimental observation of the reversed Doppler effect at an optical frequency by refracting a laser beam in a two-dimensional photonic-crystal prism^10^. Due to the shared characteristics between electromagnetic and acoustic waves, it is expected to achieve the analogous reversed Doppler effect in acoustic waves^22–27^. Recently, Lee *et al*. measured the acoustic reversed Doppler effect in one-dimensional tube with periodic thin films and side opening holes^12^. Zhao *et al*. further proposed a flute-like model of an acoustic meta-cluster with different dimensions to generate the reversed Doppler effect^15^. However, the reversed Doppler effect in an acoustic hybrid system is challenging due to the weak interaction between the acoustic meta-clusters based on traditional elements such as Helmholtz resonator and membranes.

The purpose of the work is to explore the use of hybridized metamaterial (HM) system as a means to realize reversed acoustic Doppler effect and abnormal transmission of sound waves. The idea is to couple the artificial acoustic Mie resonances of rich multipolar eigenmodes in different orders with each other efficiently^28–36^. Here we propose a HM system constructed by supercells of coupled Mie resonators, in which the traditional Mie resonator (TMR) and quadruple-channel Mie resonator (QMR) may be seen as atoms. Such system allows the simultaneous negative effective bulk modulus (${\kappa }_{eff}$) supported by the monopolar Mie resonance and the negative effective mass density (${\rho }_{eff}$) induced by the dipolar Mie resonance achieved in overlapped frequency range. The abnormal reversed Doppler effect associate with negative phase velocity are illustrated. By using such HM, the acoustic negative refraction effect and the imaging of the point source using a flat slab are successfully demonstrated.

## Results

### Theoretical model

**Figure 1:**
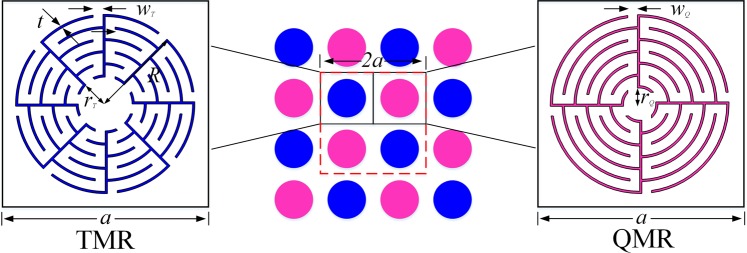
Model of the hybridized metamaterials. Schematics of the composite structure composed by TMRs (blue) and QMRs (pink) alternately patterned in square lattice. The left and right insets show the zoomed-in details of TMR and QMR unit, respectively.

Figure 1 shows the schematics of the hybridized metamaterials, in which the TMRs and QMRs are alternately patterned in square lattice. The insets show the zoom-in details of TMR and QMR units with identical lattice constant *a*, and the red-dashed square denotes a basic supercell with period 2*a*. The TMR unit (left inset) is composed of 8 identical sections with a radius *R*, and each section has a zigzag channel with a slit width ${w}_{T}$, a wall thickness *t*, and a curling number ${N}_{T}=5$; whereas the QMR unit (right inset) is composed of 4 identical sections with a slit width ${w}_{Q}$ and a curling number ${N}_{Q}=6$. These labyrinthine configurations possess extraordinary high-refractive-index relative to the background medium as the acoustic waves are forced to travel along the extended zig-zag channels, which can be determined by the section number and curling number.

**Figure 2:**
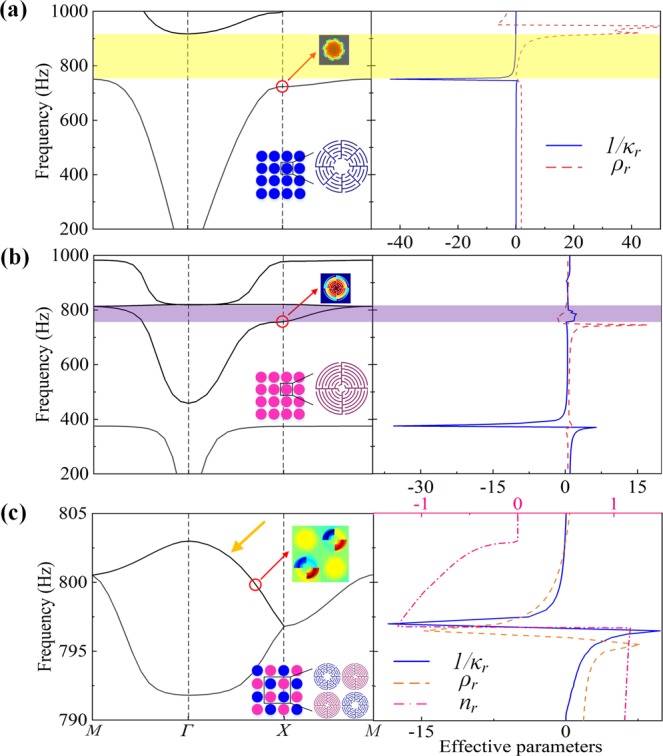
Band structure and effective parameters. Band structures (left panels) and retrieved effective parameters (right panels) of (**a**) TMR lattice, (**b**) QMR lattice and (**c**) HM in composite structure. Blue solid, yellow dashed, and pink dash-dotted curves represent the reciprocal of effective bulk modulus $1/{\kappa }_{r}$, mass density ${\rho }_{r}\,$and refraction index ${n}_{r}$, respectively. Insets: eigenmodes at the $\varGamma \,$point showing the monopolar mode of TMR lattice, dipolar mode of QMR lattice, and hybridized mode in composite structure exhibiting coupled monopolar and dipolar resonances. The shaded region represents the frequency range with $1/{\kappa }_{r} < 0$ or ${\rho }_{r} < 0$.

To characterize the performance of the proposed HM quantitatively, the band structures are numerically calculated and shown in the left panels of Fig. 2. We start with the regular square lattices as shown in the insets of Fig. 2(a,b), which are composed of identical TMR and QMR cylinders embedded in air background, respectively. Note that here we arrange the unit cells in square lattice for illustration, and HM in other typical lattices also exhibit similar characteristic due to the subwavelength scale of the unit cells, such as in triangular lattice or honeycomb lattice like graphene. In the following, we define a supercell consisting of four neighboring cylinders as the primitive cell [see the inset of Fig. 2(c)]. From the Fig. 2(a), we can see there is a bandgap in the frequency range from 750.4 to 916.8 Hz. The eigenmodes near the bandgap (see the inset) demonstrate that there is a gradient of acoustic pressure amplitude in radial direction while the amplitude is uniform in axial direction and the acoustic power is mostly concentrated inside the structure. These characteristics are similar to the spherically symmetric radial oscillation of a monopole resonator, which would cause $1/{\kappa }_{r} < 0$ over a small frequency window above resonance. The yellow shaded background represents single $1/{\kappa }_{r} < 0$ of TMR lattice, which appears in the frequency range from 750.4 to 916.8 Hz and is coincident with the bandgap in band structure. This conformity also satisfies with the monopolar resonant mode of the structure and illustrate the physical mechanism of the bandgaps. Similarly, as shown in the band structure of QMR lattice in Fig. 2(b), there exists a second bandgap in the *ΓX* direction with a bandwidth from 758.4 to 803.0 Hz (purple shaded background), which exactly falls into the bandgap of TMR lattice. This overlap is critical to achieve a double negative metamaterial.

Furthermore, to provide $1/{\kappa }_{r} < 0$ and ${\rho }_{r} < 0$ simultaneously, we employ the concept of mutual constitutive elements interaction, and construct the composite structures containing both the monopolar and dipolar resonances [see the inset of Fig. 2(c)]. Figure 2(c) demonstrates the band structure of composite structure. Note that in the overlap region of single $1/{\kappa }_{r} < 0$ and ${\rho }_{r} < 0$ mentioned above, a new pass band extending from the frequency 796.8 Hz to 803.0 Hz emerges for the particular choice of parameters used for Fig. 2(c). In addition, among the resulting branches of dispersion curves in Fig. 2(c), there is one indicated by a yellow arrow that evidently exhibits negative group velocity, since the frequency decreases with increasing wave vector *k*. To confirm the double negative material parameters, we retrieved the effective parameters including $1/{\kappa }_{r}$, $\,{\rho }_{r}\,$and ${n}_{r}$ of the composite structure, as shown in the right panels of Fig. 2. We emphasize that both the monopolar (dipolar) resonance and the single $1/{\kappa }_{r} < 0$ (${\rho }_{r} < 0$) satisfactorily maintains as the homogeneous lattice of TMR (QMR) [see Fig. 2(a,b)], despite the presence of the adjacent doped units of QMR (TMR).

Here we demonstrate the all-Mie-resonance metamaterial consisting of closely positioned Mie resonators embedded in a background medium air. The coupling between the Mie resonators could affect the acoustic response of the metamaterial, in a way similar to that observed in heterogeneous acoustic metamaterials composed by different types of resonators such as membranes and slits. We would like to note that the coupling between TMR and QMR resonators causes resonant mode hybridization and promotes the channeling of acoustic energy by coupled fields [see the eigenmodes in the inset of Fig. 2(c)], which contributes to the formation of the bands with enhanced transmission in former band gap. The supercells of coupled Mie resonators may be seen as meta-molecules, whereas the TMRs and QMRs may be seen as atoms or particles. It is therefore expected that the properties of this HM structure could be configured by the constitutive units and/or the coupling effect therein under appropriate design conditions.

### Reversed dopper effect

We demonstrate the abnormal reversed Doppler effect, which is one of the fascinating characteristics of artificial metamaterial with double-negative parameters. When sound waves propagate from air to the metamaterial, the relationship between the sound velocity and refractive indices can be expressed as ${n}_{1}{c}_{1}={n}_{0}{c}_{0}$, where *n*_1_ and *c*_1_ (*n*_0_ and *c*_0_) are the refractive indices and sound velocities of the metamaterial (air), respectively. For illustration, we assume that the sound source maintains stationary and the detector moves, the Doppler shift Δ*f* can be derived as:

1 $$\Delta f=\left(\frac{{c}_{1}\pm {v}_{d}}{{c}_{1}}-1\right)\,{f}_{0}=\left(\frac{{c}_{0}/{n}_{1}\pm {v}_{d}}{{c}_{0}/{n}_{1}}-1\right)\,{f}_{0}=\left(\frac{{c}_{0}\pm {v}_{d}{n}_{1}}{{c}_{0}}-1\right)\,{f}_{0},$$

where ${v}_{d}$ represents the velocity of the detector movement; ${f}_{0}$ is the operation frequency of sound source; the symbols + and − represent the approaching and receding movement, respectively. Note that the sign and magnitude of the Doppler shift $\Delta f$ is determined by ${n}_{1}$ at fixed ${v}_{d}$. Derived from Eq. (1), for the chosen parameters of ${v}_{d}=1\,m/s$, ${f}_{0}=799.1\,{\rm{Hz}}$ and ${n}_{1}=-\,1$, the Doppler shift $\Delta f=-\,2.3\,{\rm{Hz}}$ or 2.3 Hz when the detector moves towards or away from the source, respectively.

**Figure 3:**
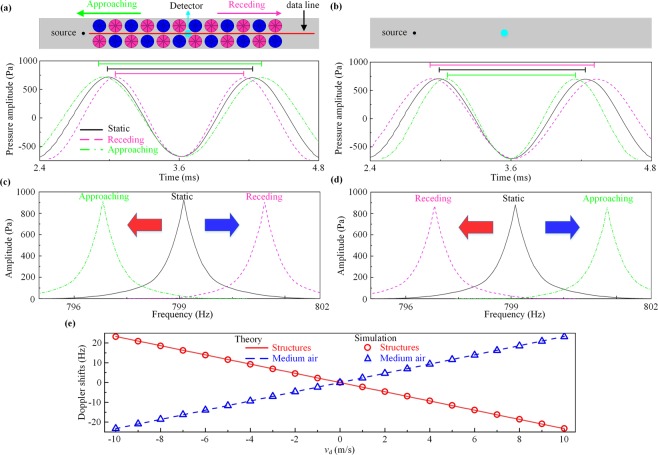
Reversed Doppler effect. Pressure waveforms in the time-domain for (**a**) abnormal reversed Doppler effect in HM and (**b**) normal Doppler effect in the background medium air for comparison across the red solid data line. The black solid, green dash-dotted and purple dashed curves denote the static, approaching and receding signal, respectively. Insets: schematic setup in which the point detector approaches or recedes from the fixed source. (**c**,**d**) Fast Fourier transformation (FFT) spectra corresponding to (**a**,**b**). The red and blue arrows represent the red and blue shifts, respectively, which are reversed in the two cases. (**e**) Doppler shift in dependence of the velocity of the detector movement ${v}_{d}$.

The above theoretical predictions can be confirmed by the computed pressure waveforms of time-domain signals in a finite thickness HM slab, which is composed by 6 layers of supercells along the propagation direction [see the inset of Fig. 3(a)]. Figure 3(a) depicts a part of the waveforms measured by the moving detector with ${v}_{d}=1\,m/s$. The green dash-dotted and purple dashed curves denote the approaching and receding signal, respectively, while the black solid curve represent the signal at static status for comparison. Note that the periodicity of the sinusoidal wave extends (shortens) from 1.251 *ms* at the static state to 1.255 *ms* (1.247 *ms*) when the detector approaches (recedes from) the fixed source. The corresponding fast Fourier transformation (FFT) spectra calculated from the full-recorded waveform are shown in Fig. 3(c), in which the peak frequency exhibits a redshift (blue shift) from 799.1 Hz at static state to 796.9 Hz (801.3 Hz) for the approaching (receding) case, that is, with a Doppler shift of $\Delta f=-\,2.2\,{\rm{Hz}}$ ($\Delta f=2.2\,{\rm{Hz}}$). However, such extraordinary phenomenon is contrary to the conventional Doppler effect in the background medium air, where the detected signals show blue shift when approaching to the source while depict redshift when receding from the source [see Fig. 3(b,d)]. Thus, we refer to these abnormal effect in HM slab as reversed Doppler effect for the sake of discrimination, and the reversed Doppler shift is in proportion to the velocity of the detector movement ${v}_{d}$ [Fig. 3(e)].

**Figure 4:**
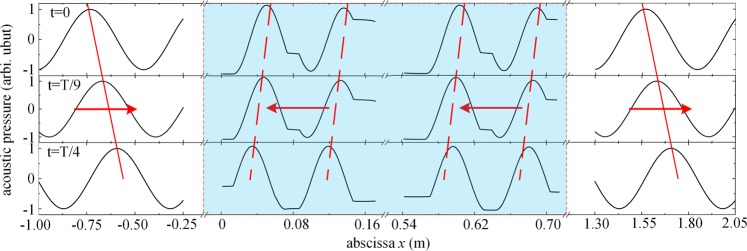
Negative phase velocities with reversed wavefronts. Three consecutive snapshots of the time-domain waveforms at $t=0$, $T/9$ and $T/4$ with a line sound source emits a series of sinusoidal acoustic plane waves with a frequency of 799.1 Hz, which transmit through a finite periodic structure with 6 supercells.

For the proposed HM slab, the reverse of the acoustic wave propagation characterized by an antiparallel phase velocity is also an indicator of negative refraction index. Figure 4 further shows the consecutive snapshots of the time-domain waveforms at $t=0$, $T/9$ and $T/4$ across the red solid data line in Fig. 3(a), which characterize the typical evolution of a series of sinusoidal acoustic plane waves with a frequency of 799.1 Hz through the HM slab. Here the blue shaded area represent the HM slab in $0 < x < 12a$, sandwiched by the background medium air in $x < 0$ and $x > 12a$. As shown by the red arrows, in regular materials outside the slab, the wavefront moves along the direction receding from the source (that is, from left to right). However, when both $1/{\kappa }_{r}$ and ${\rho }_{r}\,$are negative, we obtain that the phase velocity points toward the source (from right to left), thus, the phase velocity and energy flow are antiparallel inside an HM. In addition, by measuring the transmission wave profiles in Fig. 4, the sinusoidal waves barely decay with the propagation distance inside the metamaterial. The magnitudes of the real and imaginary parts of wave number can be estimated from the wavelength and decay rate of the wave. The real component (~14.63 m^−1^) is over 2 order of magnitude higher than the imaginary component (~0.05 m^−1^). This indicates that the negative phase velocity originates from the double negativity of the composite structure rather than dissipation effect, in which the real and imaginary components of the wave vector should be the same in magnitude instead. Thus, reversed propagation and negative phase velocity in the band of double negativity in the HM can be confirmed.

### Negative refractions

**Figure 5:**
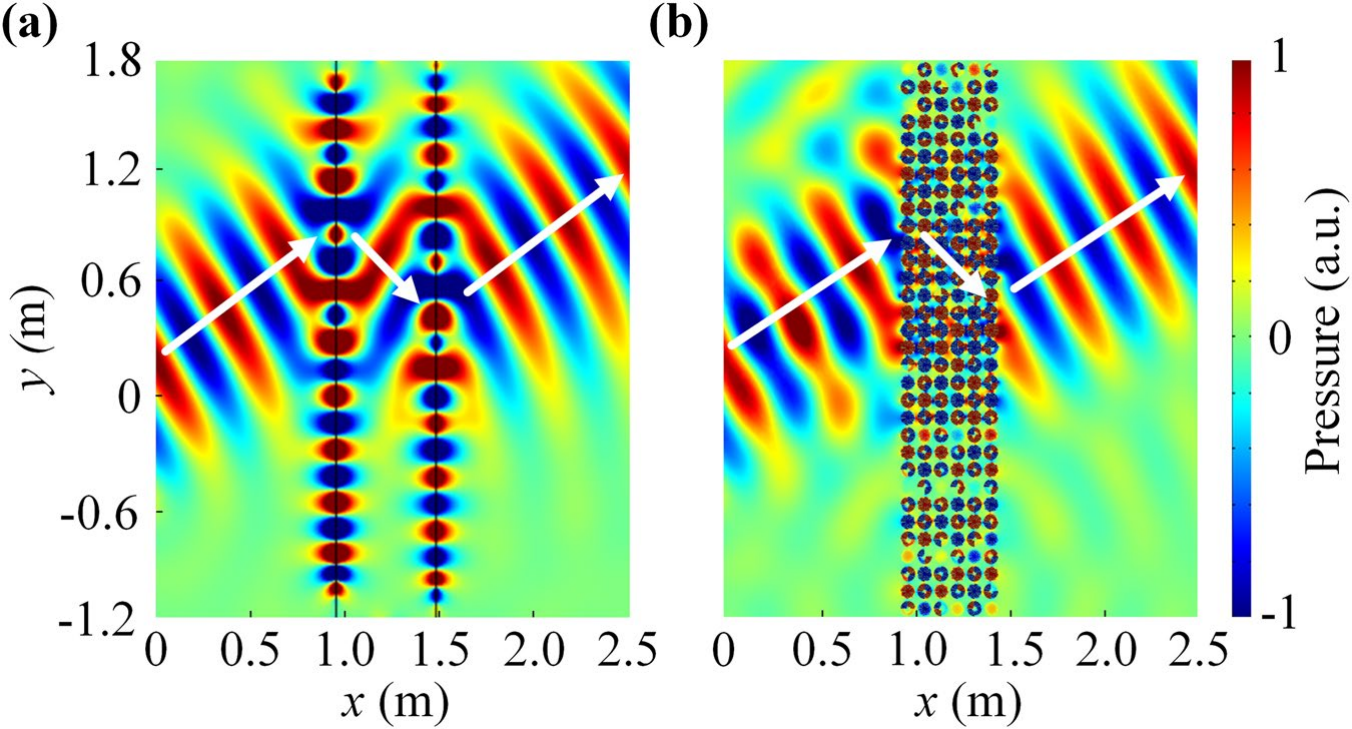
Negative refraction. (**a**) Gaussian beam of frequency 799.1 Hz incident from the left on (**a**) a rectangular effective medium with a refractive index of ${n}_{1}=-\,1$ and (**b**) a three-layer HM slab, making an angle of 30° with the vertical axis. The white arrows mark the direction of incident and refracted beams.

We have also performed simulations to see how the acoustic wave is refracted by the HM. Figure 5(a,b) plot the spatial pressure distributions at the frequency 799.1 Hz, in which we sent an incident Gaussian beam to a rectangular effective medium with a refractive index of ${n}_{1}=-\,1$ and a three-layered HM slab, respectively. As clearly seen in Fig. 5, the center of the outgoing Gaussian beam is shifted to the bottom side of the center of the incident Gaussian beam for the HM slab (see the white lines and arrows). The theoretical value and the numerical result of the refraction are 30° and 29°, respectively. The calculation results for effective medium and HM slab agree well. Refractive index of HM at 799.1 Hz determined from Fig. 5(b) is −1.02, which is very close to the theoretical value of −1 computed from Fig. 5(a). Consequently, this HM arrangement unambiguously demonstrates the behavior of negative refraction and reaffirms the effect of a “negative Snell’s Law” in acoustic waves as predicted by Veselago^14^.

**Figure 6:**
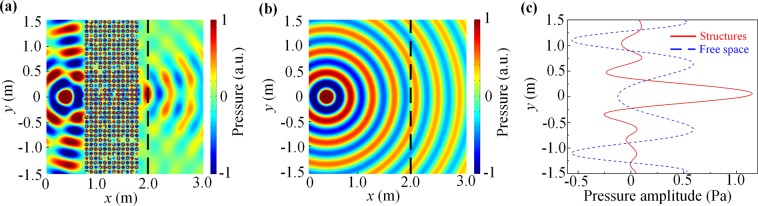
Focusing effect using a flat HM slab. (**a**) Distribution of the acoustic intensity emitted by a point source through (**a**) a six-layer HM slab and (**b**) the free space. (**c**) Corresponding pressure profiles at the black dashed line in (**a**,**b**).

Finally, we proceed to consider the focusing effect by a flat lens made of HM slab. Ideally, such an acoustic lens can focus a point source on one side of the flat lens into a real point image on the other side. To see whether the lens effect indeed exists in our HM structure, we take a 17-layer wide and 6-layer thick slab sample as an example, and a monochromatic point source radiating at the frequency 799.1 Hz is placed at a distance 6*a* (half thickness of the sample) from the left surface of the HM slab. Figure 6(a) shows the image formation of a point source through a HM slab. One can see that a clear high-intensity image is formed in the opposite side of the slab, which is in contrast to the cylindrical wave radiation pattern of the same source in free space shown in Fig. 6(b). For better comparison, the transverse distributions of the acoustic intensity at the image plane is measured along a line parallel to the right surface of the HM slab at the focus plane [black dashed lines in Fig. 6(a,b)], as shown in Fig. 6(c). The data reveals a transverse size (half width at half maximum) of the image spot about 3.6*a* in diameter, which is 3/4λ (λ being the working wavelength). Therefore, the focusing effect is realized by the flat HM slab system due to the coupled resonant nature of the metamaterial.

## Discussion

In conclusion, we proposed a composite acoustic metamaterial based on hybridized acoustic Mie resonances. By coupling the monopolar resonance in TMR and dipolar resonance in QMR with in overlapped frequency region, we realize the negative refraction index with simultaneous negative effective dynamic bulk modulus and mass density. We further demonstrate the reversed Doppler effect in the HM system with the effective and physical models. Additionally, a backward wave propagation, namely a negative phase velocity is also demonstrated. The sound waves propagating through the system display a negative refraction effect and hence the phenomenon of focusing is also achieved by a parallel-sided flat HM slab. The results show that the reversed Doppler effect is a general phenomenon in acoustic metamaterials with negative index, which may provide useful basis for future applications.

## Methods

Throughout the paper, the finite element method based on COMSOL Multiphysics with “Acoustic Pressure Frequency Domain Module” was employed for the radiated acoustic field simulations in Figs. 3–6. The hard boundary conditions were set for upper and lower sides of the impedance tube, while the perfectly matched layers were imposed on the left and right radiation boundary in order to eliminate reflections. A plane wave radiation boundary condition for the incident wave was imposed on the incident boundary. The effect of loss was not taken into account in the simulations. After introducing loss into the system, the simulation results also exhibit negative refraction in spite of weak attenuation.
